# EMT Mechanism, Lung Cancer Metastasis, and microRNA

**DOI:** 10.3389/fmolb.2021.731788

**Published:** 2021-09-30

**Authors:** Shihori Tanabe

**Affiliations:** Division of Risk Assessment, Center for Biological Safety and Research, National Institute of Health Sciences, Kawasaki, Japan

**Keywords:** lung metastasis, RNA, cancer, microRNA, epithelial-mesenchymal transition

## Introduction

Cancer metastasis occurs due to several causes such as histologic, genetic, and pathologic features. Hematogenous spread, lymphatic spread, and direct spread to pleura are the main routes of lung metastasis (Stella et al., 2019). The primary sites of the tumor may affect the route of the spread.

Many interesting theories exist regarding the origin of metastatic cells. Jamil and Kasi summarized that there would be main roots for the lung metastasis among these theories: 1). epithelial-mesenchymal transition (EMT) in which epithelial stem cells transform into mesenchymal cells, 2). stem cell origin of metastatic tumors in which tissue stem cells are the origin of metastatic cancers, 3). a concept of macrophage facilitation of metastasis in which tumor-associated macrophages contribute to tumor progression, and 4). myeloid cell origin of metastasis in which myeloid origin cells with mesenchymal properties promotes metastasis (Jamil and Kasi, 2021). The signaling pathways involved in the microenvironment include Rous sarcoma virus tyrosine kinase signaling and p38 and extracellular signal-related kinase-1 (ERK) mitogen-activated protein kinase (MAPK) signaling. Adhesion and extracellular matrix molecules are also important to establish metastatic mass.

## Pathogenesis and Involvement of Tumor-Derived Exosomes in Cancer Metastasis

### Tumor-Derived Exosomes and microRNA in EMT Mechanism and Cancer Metastasis

Jamil and Kasi highlighted the importance of tumor-derived exosomes containing proteins, DNA, RNA, and non-coding RNA. Tumor-derived exosomes may prepare the pre-metastatic niche, and establish a microenvironment at distant sites (Jamil and Kasi, 2021). Tumor-derived exosomes promote angiogenesis and EMT (Mashouri et al., 2019). TGF-β, IL-6, HIF1α, β-catenin, vimentin, casein kinase, and several EMT-inducer microRNAs (miRNAs) such as miR-301a-3p, miR-146a, miR-155, and miR-32-5p in tumor-derived exosomes are essential factors in the EMT mechanism (Mashouri et al., 2019). In the EMT mechanism, the miRNAs such as miR-200 family, miR-27a, miR-95-3p, miR-195, and miR-133 may be associated with lung metastasis. In colorectal cancer, the overexpression of miR-885-5p induced cell migration and invasion, which was associated with the development of liver and lung metastases (Jamil and Kasi, 2021). Tumor-associated macrophages, major components of tumor microenvironment associated with cancer metastasis, induce EMT for colorectal cancer migration and circulating tumor cell-mediated metastasis *via* JAK2/STAT3/miR-506-3p/FoxQ1 axis activated by IL-6 (Wei et al., 2019).

### Symptoms and Treatment of Lung Metastasis

Systematic symptoms of patients with lung metastasis are fatigue, nausea, anorexia, and weight loss, while localized symptoms include pleurisy/pleural effusion, cough, dyspnea, hemoptysis, scalp metastasis, electrolyte disturbances, Pancoast tumor, and superior vena cava syndrome (Jamil and Kasi, 2021). Several evaluations for detecting lung metastasis may include a chest X-ray, computed tomography (CT), positron emission tomography (PET), magnetic resonance imaging (MRI), and flexibltracheobronchoscopy with endobronchial ultrasound (EBUS). Jamil and Kasi indicated that specific patterns associated with different tumors on chest imaging of CT include diffuse miliary seeding, large singular metastases, calcification of metastases, and cavitation of pulmonary metastases (Jamil and Kasi, 2021). Treatment options for lung metastasis include chemotherapy where drug resistance and toxicity are the main problems, immunotherapy such as cytokine therapy, and radiation. Surgery may be an option if metastases are restricted to the lungs. The criteria for selecting patients are technical resectability, tolerable general and functional surgical risk, control of the primary tumor process, and exclusion of any further extrathoracic metastasis. A long disease-free interval between the treatment of the primary tumor and the discovery of pulmonary metastases, absence of thoracic lymph nodes metastases, and a small number of pulmonary metastases are favorable prognostic factors (Jamil and Kasi, 2021). According to Jamil and Kasi, special considerations may be needed for specific cancers in terms of surgery. Indications for removal include all residual tumors after chemotherapy and normalization of tumor markers, recurrence after chemotherapy treatment, failure to respond to chemotherapy, and partial response to chemotherapy. In case surgery is no option for pulmonary metastasis, radio frequency ablation (RFA) may be a choice. In colorectal cancer, about 20% of the patients develop lung metastasis, where RFA in combination with systemic chemotherapy may improve the median survival duration. RFA can be also applied for renal cancer and hepatocellular carcinoma (Jamil and Kasi, 2021).

### Prognosis of Lung Metastasis

The prognosis of lung metastasis varies depending on the type of tumor, molecular biomarkers, extent of the disease, or treatment modalities (Jamil and Kasi, 2021). Complications include chemotherapy side effects as oral and gastrointestinal mucositis and chemotherapy-induced peripheral neuropathy, postsurgical complication as infection, atelectasis, cardiac arrhythmia, stroke, myocardial infarction, prolonged air leak and renal failure, and radiation side effects as radiation pneumonitis and post-radiation tumors (Jamil and Kasi, 2021). Since there have emerged new potential therapeutic approaches including phospholipase A2 inhibitors, long non-coding RNAs as therapeutic or diagnostic markers (Chen et al., 2016), bergamottin inhibiting EMT (Ko et al., 2018), and frondoside A (Attoub et al., 2013), further elucidations and investigations are needed for the treatment of lung metastasis.

## Discussion

The understanding of lung metastasis with the view of EMT mechanism and RNAs would lead to the comprehensive therapy of cancers. Several kinds of RNAs including microRNAs are involved in tumor-derived exosome-mediated lung metastasis. EMT and immunosuppression of CD8^+^ tumor-infiltrating lymphocytes, which are important factors of cancer progression, are linked *via* miR-200 and ZEB1 transcription factor (Chen et al., 2014). The miRNAs which repress EMT may be potential candidates as therapeutic targets of advanced cancer. In the meantime, long non-coding RNAs inducing EMT and lung cancer cell metastasis are potential targets for therapeutical inhibition. A long non-coding RNA, JPX, and Twist1 transcription factor are up-regulated in lung cancer and induce EMT *via* inhibition of miR-33a-5p and activation of Wnt/β-catenin signaling (Pan et al., 2020). A panel of miRNAs, miR-193a-3p, miR-210-3p, and miR-5100, in hypoxic bone-marrow stem-cell-derived exosome promotes lung cancer metastasis *via* STAT-3 induced EMT, which may be a candidate biomarker for cancer metastasis (Zhang et al., 2019). A multi-layer regulatory network including TGF-β, Wnt, growth factors, Notch, and hypoxia is involved in the regulation of EMT-related transcription factors, where several miRNAs such as miR-34, miR-200s, and miR-205 regulate the expression of transcription factors (Lu and Kang, 2019). EMT mechanism is essential in cancer metastasis, while the excess EMT may lead to cell death (Lu and Kang, 2019). Furthermore, although EMT induces cancer migration, the EMT mechanism itself is not sufficient for the whole process of cancer metastasis. In addition to the EMT mechanism, mesenchymal-epithelial transition (MET) is necessary for cancer colonization (Aiello and Kang, 2019). Regulation of miRNAs in EMT and MET are involved in cancer metastasis (Aiello and Kang, 2019). The potential of RNAs as therapeutic targets or biomarkers in EMT-induced cancer metastasis would further increase. It would be essential to reveal EMT mechanism and cancer metastasis from the viewpoint of RNA regulation.
